# Cross-species characterisation of microglial morphology and proliferation during ageing

**DOI:** 10.1007/s11357-026-02254-3

**Published:** 2026-04-24

**Authors:** Laura M. Carr, Angus McNamara, Shannon M. Stuckey, Isabella M. Bilecki, Sanam Mustafa, Renée J. Turner, Lyndsey E. Collins-Praino

**Affiliations:** 1 School of Pharmacy and Biomedical Science, Adelaide University, Adelaide, SA Australia; 2 Davies Livestock Research Centre, Adelaide University, Roseworthy, SA Australia; 3 Australian Research Council Centre of Excellence for Nanoscale Biophotonics, The University of Adelaide, Adelaide, SA Australia

**Keywords:** Microglia, Ageing, Cross-species, Neurodegeneration

## Abstract

By 2050, one-fifth of the world is expected to be over 60, and the prevalence of age-related neurological conditions is predicted to increase dramatically. Aged animals are currently underutilised in neurological research, leading to a gap in knowledge about the contribution of biological age to the pathophysiology of age-related neurological conditions. Additionally, it is unclear whether age-related changes differ across species used in preclinical models, and how these differences may compare to the aged human brain. Understanding these points is critical for successful translation of findings from preclinical studies to the human context. The current study presents a cross-species characterisation of microglia, the key regulator of the brain’s immune response, during ageing. Microglial number, proliferation and morphology were assessed in archival tissue from Sprague Dawley rats (males; 3 to 18 months old) and Merino sheep (males and females; 1 to 6 years old), with these two species selected for their relevance to preclinical modelling of neurological disease. Increased numbers of proliferating microglia were observed in the cortex, hippocampus and portions of the striatum in both species. This proliferation declined at the oldest timepoint assessed (i.e. 18 months old) in rats, a pattern not seen in the sheep. Total microglial number was largely unchanged with age in the rat brain; however, in sheep, the number of microglia decreased significantly in the dentate gyrus in older animals. Notably, microglia in 18-month-old rats were larger in all regions, but changes in branching were observed exclusively the striatum. Similarly, in sheep, morphological changes were localised to the striatum, with increased cell and soma size in the caudate nucleus, and increased cell size and process length in the putamen. These changes suggest a shift away from homeostasis in the cortex and hippocampus and towards a semi-ramified morphology in the striatum in late middle adulthood that is largely conserved across these two species. Nevertheless, the age of the oldest animals here equates to only ~ 60 years old in humans, rather than reflecting an aged human population. Thus, future work is needed to understand how species-specific differences continue to evolve in older age.

**Supplementary Information:**

The online version contains supplementary material available at https://doi.org/10.1007/s11357-026-02254-3.

## Introduction

Neurological diseases currently affect millions of people and are a significant contributor to disability and mortality worldwide. In 2021, despite pandemic conditions increasing the effect of COVID-19 related deaths, stroke and Alzheimer’s disease (AD) remained among the top 10 leading causes of death globally, occupying 3rd and 7th place, respectively [1]. Increasing age is the leading non-modifiable risk factor for stroke [2] and is the number one risk factor for developing AD [3]. With one-fifth of the global population expected to be over 60 years old by 2050 [4], the burden of these age-related neurological conditions is expected to increase dramatically. Indeed, the effect of population ageing on another age-related neurological disorder, Parkinson’s disease (PD), is already evident. In 2016, PD was the fastest growing neurological disorder [5], with the crude prevalence rate increased by 74% compared to 1990 [5]. Notably, the age-standardised prevalence during this time increased by 22%, demonstrating that, while other factors may also contribute to the increasing PD prevalence, our ageing population is a major factor. Despite their significant burden, however, neurological diseases remain difficult to treat and translation of potential treatments to the clinic is notoriously slow, with many drugs failing to demonstrate the benefits seen in preclinical models when assessed in clinical trials [6]. To improve translation rates and successfully develop effective treatments for age-related neurological conditions, it is critical that experimental models incorporate the aged phenotype, in order to best reflect the pathophysiological conditions that occur in disease states [7, 8]. To do so, it is important to understand how key hallmarks of ageing, such as chronic inflammation [9], are altered throughout the lifespan in animal models commonly utilised in preclinical research.

Mice and rats comprise the majority of animal models utilised in neurological disease research [10] and are preferred not only for the logistical benefits, including their small size, ease of handling, low cost and short lifespan [11], but also because there are well-established behavioural batteries and techniques for genetic manipulation available for these species [11]. Nevertheless, rodent models present with key neuroanatomical distinctions when compared to humans, potentially impacting findings [12]. For example, rodent brains are lissencephalic [13], with a smaller proportion of white matter compared to humans [12]. This is important in the context of neurological injury, where there is a high clinical incidence of white matter involvement [12]. Furthermore, the rat cortex is much thinner, with a less complex cortical architecture [14] and connectivity [15] than that of humans [16], and the overall size of the rat brain (1–2 g) is several orders smaller than that of humans (1.3–1.4 kg) [17]. Comparatively, many large animals, such as sheep and pigs, have gyrencephalic brains with a more comparative structure [17], weight [17] and proportion of white matter [12] to that seen in humans. Therefore, the utilisation of these large animal models is likely to enhance the translatability of results and better reflect human condition [12, 17, 18].

Species-specific differences are observed in both peripheral and central immune responses between rodents and humans. Humans and mice are known to differ in their peripheral immune cell populations, with humans having neutrophil rich immune populations and mice favouring leukocytes [19]. Interestingly, cells involved in the central immune response seem to show more similarities, with neuroinflammatory models built using Biological Expression Language demonstrating a high degree of similarity between species when investigating the interactions of microglia and astrocytes (62% similar interactions, with 28% of interactions seen only in humans, and not in mice) [20]. Notably, however, only 27% of cytokine interactions were conserved across species, with 58% unique to humans [20]. In comparison, less is known about how the ovine immune response compares to that seen in humans, particularly within the central nervous system (CNS). One study compared immune factors in blood samples collected from adult female sheep (~ 3 years old) and humans (~ 28 years old) following ex vivo stimulation with either 1 µg/ml lipopolysaccharide (LPS) or 10 µg/ml monophosphoryl lipid A. It was found that gene expression of key pro-inflammatory factors, including interleukin-6 (IL-6), tumor necrosis factor (TNF) and nuclear factor kappa B (NF-κB), was upregulated in response to stimulation in both species [21]. While this suggests that the peripheral immune response in sheep shares similarities with that seen in humans, further research is needed to understand how the central immune response compares between species.

This response is particularly relevant in light of the critical role that neuroinflammation plays across multiple age-related neurological diseases (for example, see reviews [22, 23]). Neuroinflammation is characterised by chronic and sustained activation of immune cells in the brain, with microglia in particular shown to be key for driving the initiation of this response [24]. Under physiological conditions, microglia are motile cells, routinely surveying the CNS microenvironment for tissue damage or potential pathogens [25]. As part of their homeostatic phenotype, microglia display a ramified structure. The complexity of these ramifications has been observed to vary slightly between species, with humans showing less complex morphology than other primates, but more than other high-order vertebrates, such as the whale [26]. Upon perceived immunological threat, microglia shift towards a phagocytic phenotype, traditionally characterised by an amoeboid shape, with thicker and shorter processes [27]. Such a morphological shift has been reported to occur across multiple species, including humans [28], mice [29, 30], rats [31], marmosets [32] and sheep [33, 34]. While diversities in microglial morphology across species have previously been characterised [26] and reviewed [35], species-specific differences in microglial functions throughout the lifespan, have not been widely investigated.

Functionally, aged microglia have been described as having a hyper-inflammatory phenotype, with studies reporting a heightened response to inflammatory stimuli. For example, in aged (22–24 months old) C57BL/6 male mice, microglia showed upregulation of soluble IL-6 receptors compared to mice at 3 to 5 months old [36] and upregulation of the pro-inflammatory cytokines IL-1β and IL-6, as well as major histocompatibility complex II (MHCII), a marker of immune activation [36]. These changes toward a state of microglial priming appear to occur in spite of maintaining a stable population [37, 38]. In line with this, a comparison of microglial proliferation in humans and mice found largely no changes in proliferation of microglia in mice or in post-mortem human tissue (with the exception of BrdU-labelled proliferating cells in the dentate gyrus (DG) in 18–24-month-old mace compared to 4–6-month-old mice) [37]. This suggests that baseline proliferation of microglia is stable in both species and changes in functional phenotypes may occur in long-lived resident microglia, rather than through replacement of the microglial population. To our knowledge, however, only this single study has been conducted examining microglial ageing across species and was focused exclusively on microglial proliferation, rather than on functional changes.

As such, more detailed microglial ageing investigations across multiple brain regions in species commonly used for pre-clinical neurological disease models is essential to guide informed use of appropriate animal models and drive improved clinical translation for neurological disorder treatments. Thus, the present study aimed to investigate changes in microglial number, proliferation and morphology in the context of ageing in rat and sheep cohorts. These changes were assessed within multiple neuroanatomical regions known to demonstrate vulnerability in neurological diseases.

## Methods

### Ethics

In line with the aim to improve the ethical use of animals in testing according to the 3R principles [39], all tissue used within this study was derived from archival sources. We used all tissue samples available and viable from these archival sources in the current analysis. Both the rat and sheep datasets contained tissue that had been stored for similar lengths of time. Ethics approval and cohort details are shown in Table 1. All experiments were performed in accordance with the Animal Welfare Act (1985) and the National Health and Medical Research Council (NHMRC) *Australian Code for the Care and Use of Animals for Scientific Purposes* (8th edition, 2013), with reporting in accordance with the ARRIVE guidelines [40, 41].

**Table 1:** Details of ethics approval and cohort details for murine and sheep datasets

Approving body	Ethics approval	Dataset	Ages	Number of animals		
				**Male**	**Female**	**Total**
The University of Adelaide Animal Ethics Committee	M-2015-11b	Young rat	3-month-old (3 months)	7	0	30
	M-2019-103	Adult rat	13-month-old (13 months)	8	0	
			15-month-old (15 months)	8	0	
			18-month-old (18 months)	7	0	
The South Australian Health and Medical Research Institute Animal Ethics Committee	SAM402.19	Sheep	1–1.5 years old (1–1.5 years)	8	8	42
			3–3.5 years old (3–3.5 years)	8	8	
			5–6 years old (5–6 years)	0	8	

### Animal details

For the rat dataset, sham tissue was sourced from previously conducted stroke studies. Male Sprague–Dawley rats (aged 3–18 months; male *n* = 30, weight 370 ± 20.37 g) were housed under conventional laboratory conditions with a 12-h light/dark cycle and access to standard rodent chow and water ad libitum. Tissue for the sheep dataset was sourced from a previously conducted lifespan study, where naïve animals that were not subjected to any experimental conditions beyond anaesthesia and tissue collection. Merino adult sheep (aged 1 to 6 years; male wethers *n* = 18, weight 65.2 ± 12.4 kg; female ewes *n* = 24, weight 58.4 ± 7.3 kg) were obtained and housed at the South Australian Health and Medical Research Institute (SAHMRI) Preclinical, Imaging and Research Laboratories (PIRL) 2-weeks prior to study involvement to enable habituation. Due to the marked sex imbalance in the oldest sheep cohort (8F:0 M), sexes were pooled to avoid biased statistical comparisons and maintain statistical power. Sex-stratified data are provided in Supplementary Table S1 and Fig. S1 for transparency.

### Rat tissue collection and processing

All animals underwent anaesthesia and tissue perfusion via direct cardiac perfusion and exsanguination prior to brain tissue collection. Briefly, animals were anaesthetised (5% inhalational isoflurane with 2L/min normoxic air mix) in the supine position and a thoracotomy was performed horizontally below the sternum. Transcardial perfusion was then performed with 100 µl of heparin (5,000 IU/ml; Pfizer, USA), followed 10% neutral buffered formalin (NBF) (POCD Healthcare, Australia). Formalin was pumped at a consistent rate (12 rpm gradually increased to 24 rpm) for 8 min or until the perfusate ran clear, following which formalin perfusion was ceased. To reduce perfusion artifacts, animals were left intact for 30 min prior to decapitation and brain removal.

After decapitation and brain extraction, brain tissue was submerged in 10% NBF (POCD Healthcare, Australia) for 10 days and processed within 14 days of extraction. 2 mm thick coronal slices were prepared and processed through changes of ethanol (75–100%), xylene and paraffin over a 16-h cycle, before being embedded in paraffin wax. Using the rat brain atlas for reference [42], 5 µm sections were cut using a microtome at the levels of the striatum and hippocampus to obtain key regions of interest (Table 2), mounted on glass histology slides and stored at room temperature until histological staining was performed.

**Table 2:** Regions of interest in each dataset. Hippocampal and striatal regions were further split into sub regions in sheep tissue where delineations between regions were well defined

Rat	Sheep
Cortex	Cortex
Hippocampus	Dentate gyrus (DG)
	Hippocampus proper (HP)
Striatum	Caudate nucleus (CN)
	Putamen (PUT)

### Sheep tissue collection

Fasted animals were weighed and anaesthetised via intravenous injection of ketamine (1 ml/20 kg, Ceva Animal Health Pty Ltd, Australia) and diazepam (2 ml/20 kg, Ceva Animal Health Pty Ltd, Australia), intubated and anaesthesia maintained using inhalational isoflurane (2–3%, Provet Pty Ltd, Australia). Heparin (25,000 IU, Pfizer, USA) was administered intravenously into the jugular vein and allowed to circulate for 5 min before the start of the procedure. Animals were placed in the supine position, while a midline incision overlying the trachea was performed, the carotid arteries were isolated and PE tubing lines were inserted rostrally into both carotid arteries. Cold Tris-saline (2L) was pumped through the carotid lines at 120 mmHg and both jugular veins were incised to allow exsanguination. The brain was then removed and sectioned into 10 mm coronal slices using a custom ovine brain matrix for embedding and histological staining. Brain slices were submerged in 10% NBF (POCD, Healthcare, Australia) before being processed through changes of ethanol (75–100%), xylene and paraffin over a 69-h cycle and embedded in paraffin wax. Using a sheep brain atlas [43], 5 µm thick sections of single hemispheres (left or right randomly allocated within age and sex groups) were prepared using a microtome from embedded tissue at the levels of the striatum and hippocampus to obtain key regions of interest (Table 2), mounted on microscope slides and stored at room temperature until undergoing histological staining.

Analysis was focused on the cortex, striatum and hippocampus. The cortex was of particular interest due to the divergent neuroanatomical structure between species described above and its central role in higher-order processing. The striatum contains a dense microglial population [44], and in addition to being implicated in age-related neuropathology, [45] it is highly vulnerable to the effects of neuroinflammation [46, 47]. Similarly, the hippocampus is particularly susceptible to neuroinflammatory mechanisms [48, 49] and is one of the most commonly studied structures in the literature on microglial ageing [50, 51], enabling the comparison of findings to prior work. In the sheep cohort, the striatum and hippocampus were further divided into sub-regions as delineation of regions was well defined in this tissue. Table 2 presents a summary of the regions analysed within each dataset and examples of the regions captured are presented in Supplementary Fig. S2.

### Immunofluorescence and imaging

Immunofluorescent (IF) staining was performed on rat and sheep brain tissue. Briefly, slides were deparaffinised in xylene and washed in 100% ethanol. Following this, slides were washed twice in phosphate buffered saline (PBS) and heat-induced antigen retrieval was conducted (in citrate solution). Slides were then washed in 0.1% triton-X-100 (Sigma-Aldrich T8787) and PBS (PBS-T) and blocked in 3% normal goat serum (NGS, Gibco #16,210,064) for 1 h at room temperature before being incubated overnight at room temperature in the primary antibodies (see Table 3 for all antibody details). On the following day, slides were washed three times in PBS and incubated for 1 h at room temperature in secondary antibodies protected from light. Following this, slides were then washed twice in PBS and incubated with DAPI (Sigma-Aldrich #D9542) for 5 min before being mounted on coverslips using Fluoromount-G mounting medium (Thermofisher, 00-4958-02). Slides were left to dry at room temperature protected from light for at least 2 h before being cleaned and imaged using a Zeiss AxioScan.Z1 at 20 × magnification (Zeiss Microscopy).

**Table 3:** Antibody concentrations used in each dataset

Antibody	Host species	Source	Rat concentration	Sheep concentration
*Primary antibodies*				
Ionized calcium binding adaptor molecule 1 (IBA1)	Mouse	Thermofisher: MA5-27726	1:1000	1:1000
Ki67	Rabbit	Abcam: ab15580	1:1000	1:1000
*Secondary antibodies*				
Alexa Fluor 647 Goat anti-mouse IgG (H + L)	Goat	Thermofisher: A32728	1:400	1:400
Alexa Fluor 488 Goat anti-rabbit IgG (H + L)	Goat	Thermofisher: A32731	1:400	1:400
DAPI	N/A	Sigma-Aldrich: D9542	1:10,000	1:10,000

### Image analysis

#### Cell counting and positive Ki67 detection

**Fig. 1:**
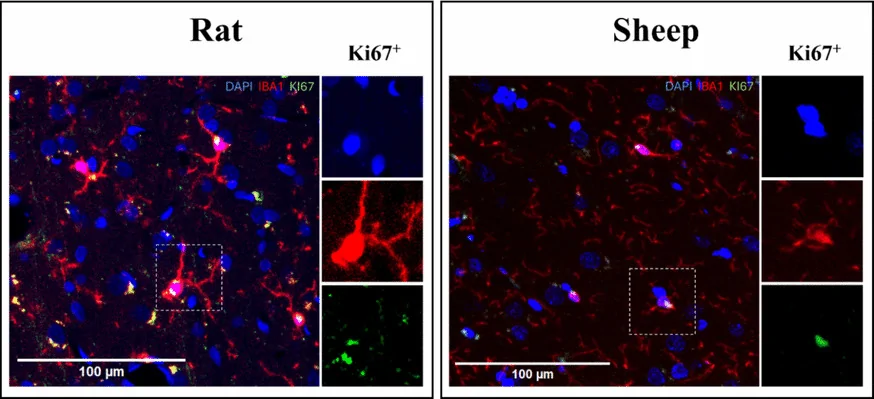
Representative images of immunofluorescent staining taken from the cortex of the oldest rat (18 months) and sheep (5–6 years) brains. Microglia were counted by identifying colocalised DAPI and IBA1. Microglia were classified as Ki67 + when DAPI, IBA1 and Ki67 signals were present. Number of total and percentage of Ki67 + microglia in ROIs were counted manually. Scale bar = 100 µm

Microglial cell counts were conducted manually in QuPath (v0.4.4) [52] on IBA1 stained images by an assessor blinded to age groups. Colocalisation of DAPI and IBA1 was used to identify microglia within annotated regions of interest in rat and sheep tissue. Microglia positive for Ki67, identified when Ki67 signal was visible in the cell soma, were also counted in these areas. IBA1 stained microglia in the cortex of the oldest animals are shown in Fig. 1, with Ki67 positive (Ki67^+^) cells indicated. Supplementary Fig. S3 displays representative images for other regions in rat and sheep brains. Cells/mm^2^ (total cell count/area analysed) and percentage of Ki67^+^ microglia (Ki67 count/total count × 100) were calculated for analysis.

#### Single-cell morphological analysis

**Fig. 2:**
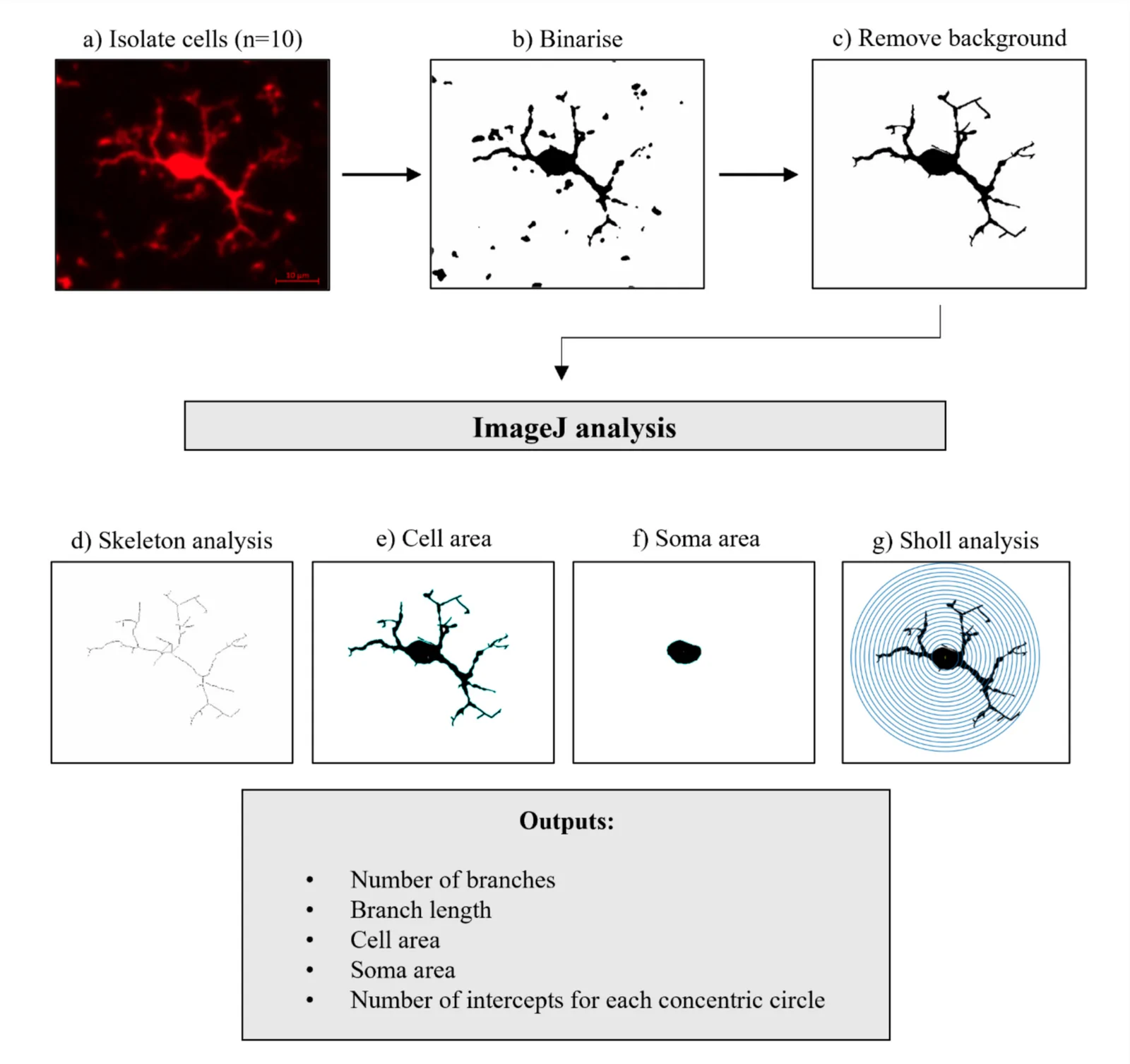
Microglial morphological analysis using ImageJ. **a** Ten cells in each region were isolated for analysis. **b** Images were binarised and **c** background pixels were removed. **d** Cells were then skeletonised to obtain number of branches and average branch length. **e** Whole cell and **f** soma area were measured. **g** Sholl analysis was conducted to obtain the number of times microglial branches intercepted each concentric circle

Morphology of microglia was assessed using the IBA1 stained images. For each animal, 10 cells from each region were randomly extracted for morphological analysis using ImageJ [53]. While prior studies utilise 25–30 cells per animal [54], given the large number of animals and number of regions included for analysis in the current work, 10 cells were selected at random to provide representative coverage of microglial morphology within each region, while maintaining practical feasibility for manual tracing analyses. Individual cell measurements were used in analysis, rather than an average per animal, as described below for each metric. Briefly, cells were isolated to individual images and converted to binary (Fig. 2a and b). Background pixels were then removed and sections of the cell that had been fragmented in the processing stages were connected by a single experimenter using the paintbrush tool and the original image for reference (Fig. 2c). This method was chosen as opposed to a static threshold due to variations in staining across the slide causing inconsistent binarization of microglia and is consistent with previously published methods in the area of microglial morphological analysis [55]. Four copies of the image were saved, one for each of the analyses illustrated in Fig. 2. For skeleton analysis (Fig. 2d), binary images were skeletonized and analysed using the AnalyzeSkeleton plugin [56] to obtain an individual number of branches per cell and mean branch length per cell. Cell area (Fig. 2e) was assessed using the second binary image on ImageJ. Soma area (Fig. 2f) was assessed by isolating the soma within the binary image of the cell and running area analysis on ImageJ. Each cell measurement for area (total and soma) was utilised in statistical analysis as below. Sholl analysis was conducted on ImageJ using the Sholl analysis plugin [57]. The centre point and maximum radius of each cell was first defined by the assessor using the line tool in ImageJ (Fig. 2g). From this point, concentric circles were drawn at 2.5 µm increments starting 5 µm from the centre point. The number of branches intercepting each concentric circle was counted for a measure of branching complexity.

### Statistical analysis

For total microglia and Ki67 positive microglia analysis, outliers with a Q value of 1% were identified in GraphPad Prism version 10.0.0 for Windows (GraphPad Software, San Diego, California USA) using the Robust Regression and Outlier Removal (ROUT) method [58] and were removed. All subsequent statistical analysis was conducted in R (version 1.4.1717) [59], sensitivity analysis containing the outliers is presented in Supplementary Figure S4. Data from manual counts were analysed using a one-way analysis of variance (ANOVA), with Tukey post hoc, to investigate the effect of age within each region of the brain. Single-cell morphological data did not undergo outlier removal using the ROUT method and were analysed in R (version 1.4.1717) [59] using linear mixed effects models in the *lme4* package [60]. As the variable of interest was age, animal age groups acted as the fixed effect, with the youngest cohort for each species (rat: 3 months; sheep: 1–1.5 years) acting as the reference. A random intercept for animal ID was included to account for individual variability in the data, and for Sholl analysis, an additional random intercept of distance was also included to account for repeated measures across distances. These models were based on a similar published analysis on single-cell microglia data [54], where distance from cell body was included as a continuous variable. In the present study, given that random effects are traditionally categorical, distance from cell body was operationalised as increments of 10 µm. For all analyses, the significance level was set at *p* < 0.05. As per previous recommendations for exploratory, hypothesis-generating work such as that presented here, *p*-values were not adjusted for multiple comparisons [61, 62].

## Results

### Microglial numbers in the rat are relatively stable with age, but proliferation increases dramatically up to 15 months of age before declining

**Fig. 3:**
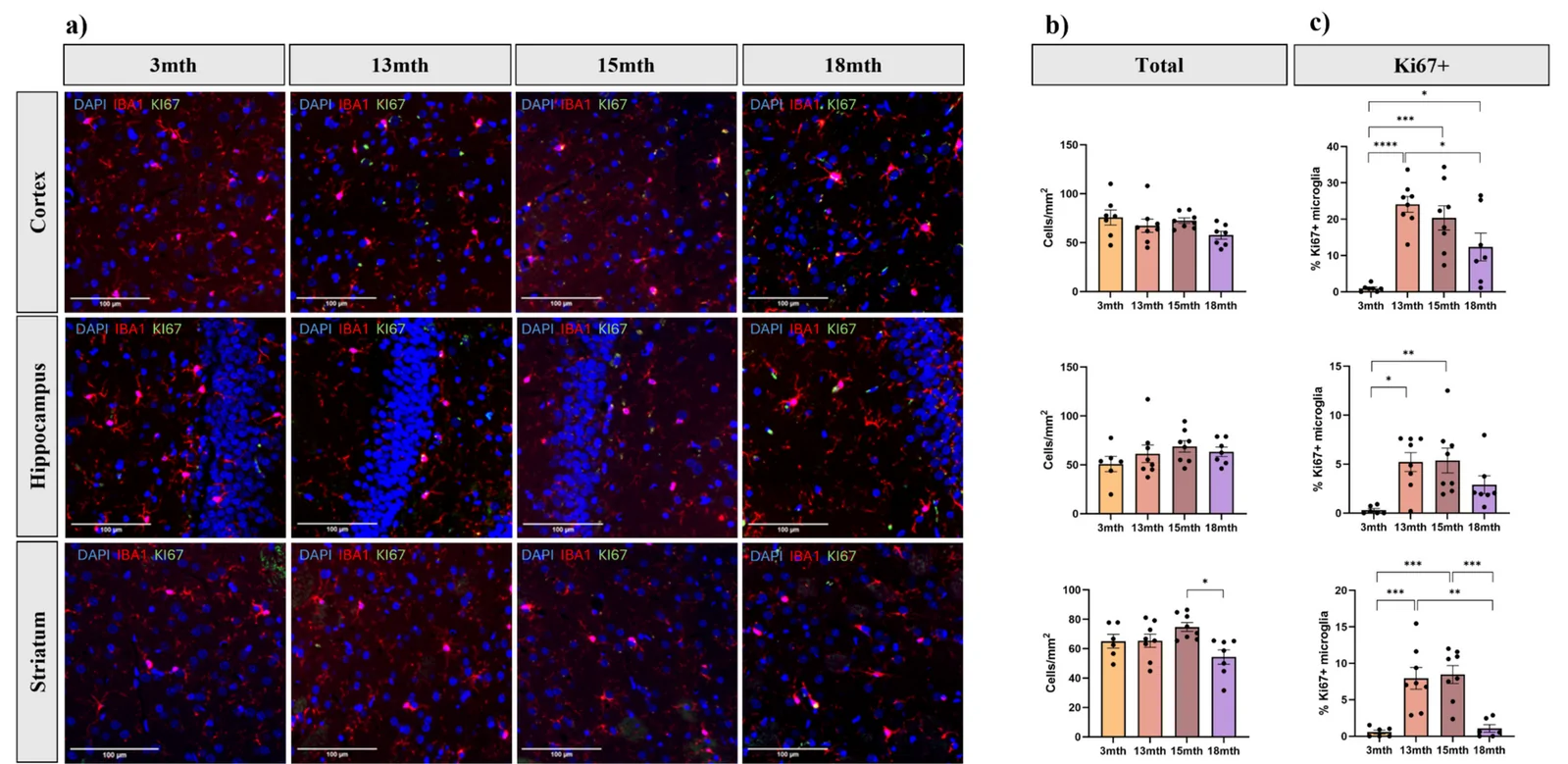
Microglia counts in the rat brain. **a** Representative merged images of microglia (red) in different regions of the rat brain at each age. IBA1 + cells with a strong Ki67 signal (green) were classified as Ki67 + microglia. Individual channels are shown in Supplementary Figures S5-S7. **b** Total number of microglia and **c**) percentage of Ki67 + microglia were manually counted and graphed as mean ± SEM. Significant differences were determined by one-way ANOVA and Tukey post hoc testing. * = * p* < 0.05, ** = * p* < 0.01. Scale bar = 100 µm

Total microglial counts and the percentage of Ki67^+^ microglia were analysed using one-way ANOVA to assess differences across ages. Representative images from each region and age group are presented in Fig. 3a. No differences in microglial number were observed in the rat cortex (*F*(3,26) = 1.874, *p* = 0.159) or hippocampus (*F*(3,24) = 2.143, *p* = 0.121), but a significant effect of age was detected in the striatum (*F*(3,25) = 3.958, *p* = 0.019) (Fig. 3b). Tukey post hoc testing showed that the total number of microglia decreased at 18 months compared to at 15 months (74.80 ± 8.54 vs 54.29 ± 12.88, *p* = 0.01). Nevertheless, no overall differences between total microglia counts at 3 months and 18 months were detected (65.12 ± 11.45 vs 54.29 ± 12.88, *p* = 0.349).

The percentage of the microglia population positive for Ki67 in rat tissue was altered in the cortex (*F*(3,26) = 13.44, *p* = < 0.0001), hippocampus (*F*(3,25) = 5.36, *p* = 0.005) and striatum (*F*(3,24) = 13.68, *p* = < 0.0001). Tukey post hoc testing revealed significant increases in the percentage of Ki67^+^ microglia in the cortex (13 months vs 3 months (24.08 ± 6.11 vs 0.95 ± 0.95, *p* = < 0.0001), 15 months vs 3 months (20.39 ± 9.4 vs 0.95 ± 0.95, *p* = < 0.001), 18 months vs 3 months (12.36 ± 10.08 vs 0.95 ± 0.95, *p* = < 0.0001)). At 18 months however, the percentage of proliferating cells began to decline, with a significant decrease detected compared to 13 months (12.36 ± 10.08 vs 24.08 ± 6.11, *p* = 0.029). In the hippocampus, increases in proliferating cells were seen at both 13 months and 15 months, compared to 3 months (13 months: 5.21 ± 2.73 vs 0.30 ± 0.41, *p* = 0.011, 15 months: 5.37 ± 3.58 vs 0.30 ± 0.41, *p* = 0.008). No overall change was seen between 3 and 18 months in the hippocampus (0.30 ± 0.41 vs 2.89 ± 2.41, *p* = 0.322). In the striatum, there was an increase in the percentage of Ki67^+^ cells at both 13 months and 15 months compared to 3 months (13 months: 7.94 ± 4.2 vs 0.57 ± 0.66, *p* = < 0.001, 15 months: 8.45 ± 3.48 vs 0.57 ± 0.66, *p* = < 0.001), an effect not seen at 18 months (1.08 ± 1.26 vs 0.57 ± 0.66, *p* = 0.991). As was observed in the cortex, in the striatum at 18 months, there was a decrease in the percentage of Ki67^+^ cells in the striatum compared to both 13 months (1.08 ± 1.26 vs 7.94 ± 4.2, *p* = 0.001) and 15 months (1.08 ± 1.26 vs 8.45 ± 3.48, *p* = < 0.001) (Fig. 3c).

### Microglial numbers in sheep decline with age in subcortical regions, while proliferation increases across multiple brain areas

**Fig. 4:**
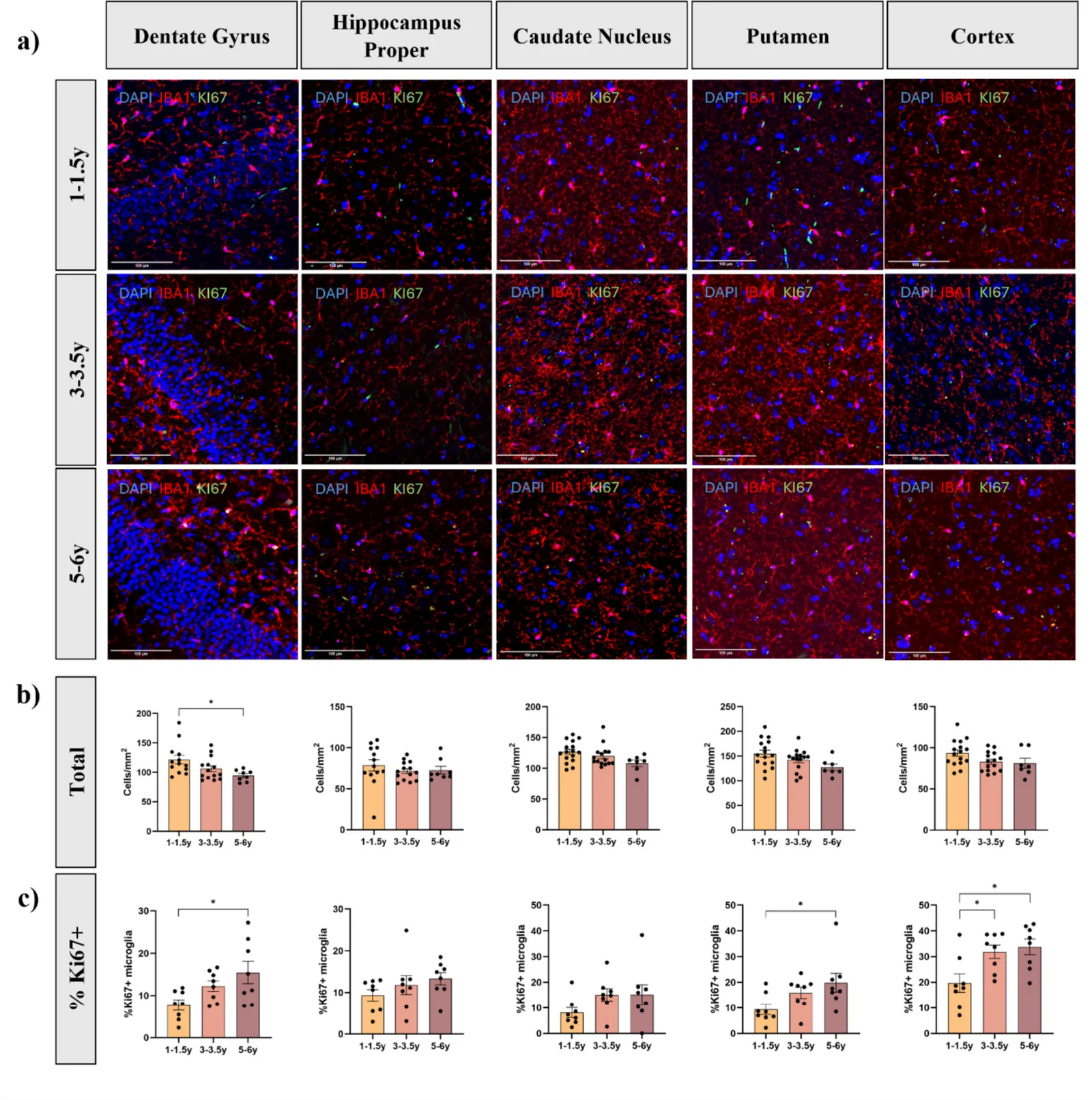
Microglia counts in the sheep brain. **a** Representative merged images of microglia (red) in different regions of the sheep brain at each age. IBA1 + cells with a strong Ki67 signal (green) were classified as Ki67 + microglia. Individual channels are shown in Supplementary Figures S8-S12. **b** Total number of microglia and **c** percentage of Ki67 + microglia were manually counted and graphed as mean ± SEM. Significant differences determined by one-way ANOVA and Tukey post hoc testing. * = * p* < 0.05, ** = * p* < 0.01. Scale bar = 100 µm

Total number of microglia and percentage proliferating microglia in sheep brain tissue was analysed using one-way ANOVA. Representative images from each region and each age group are displayed in (Fig. 4a). A significant effect of age was detected on the total number of microglia (Fig. 4b) in the DG (*F*(2,32) = 4.471, *p* = 0.019), but not in any other region (HP (*F*(2,32) = 0.564, *p* = 0.57), CN (*F*(2,35) = 3.031, *p* = 0.06), PUT (*F*(2,35) = 3.221, *p* = 0.052), cortex (*F*(2,35) = 2.857, *p* = 0.07)). Post hoc testing showed a significant decrease in total number of microglia in 5–6-year sheep compared to 1–1.5-year sheep in the DG (94.49 ± 9.59 vs 116.35 ± 19.31, *p* = 0.015). It should be noted that the caudate nucleus, putamen and cortex all showed a decrease in total number of microglia in 5–6-year sheep, although this failed to reach statistical significance.

Analysis of proliferating microglia in sheep tissue (Fig. 4c) revealed changes with age in the cortex (*F*(2,21) = 6.104, *p* = 0.008), the DG (*F*(2,21) = 4.483, *p* = 0.023) and the PUT(*F*(2,21) = 3.813, *p* = 0.038), but not in the HP (*F*(2,21) = 1.338, *p* = 0.284) or CN (*F*(2,21) = 1.935, *p* = 0.169). Tukey post hoc testing revealed a significant increase in proliferation at both 3–3.5 years (19.72 ± 10.10 vs 31.9 ± 7.4. *p* = 0.028) and 5–6 years (19.72 ± 10.10 vs 33.78 ± 8.5, *p* = 0.011) compared to 1–1.5 years in the cortex, as well as at 5–6 years (7.74 ± 3.43 vs 15.44 ± 7.46, *p* = 0.018) compared to 1–1.5 years in the DG. Additionally, in the PUT a significant increase in proliferating microglia was seen at 5–6 years compared to 1–1.5 years (9.37 ± 5.69 vs 19.84 ± 10.23, *p* = 0.032) (Fig. 4c).

### Middle-aged rat microglia display increases in soma size and area across all brain regions, with changes in branching confined to the striatum

Linear mixed effects models were employed to understand the effect of age on microglial morphology in regions of interest within the rat brain. Results from models are presented in Table 4 for area and skeletal analyses and Table 5 for Sholl analysis. Models used the 3-month group as a baseline category and included a fixed effect of age and random intercepts for animal ID, and in Sholl analysis, distance, which was operationalised as increments of 10 µm. For ease of reporting, estimates and confidence intervals are presented only in the relevant tables.

**Table 4:** Linear mixed effects modelling of microglial morphological analyses in rat brain tissue. Individual microglia were isolated from each animal (*n* = 10 per region) and analysed on ImageJ as described in Fig. 2 to obtain cell area, number of branches, mean branch length per cell and soma area. Individual cell data were analysed using linear mixed effects modelling within the cortex, hippocampus and striatum. Models contained a fixed effect of age, and a random intercept of animal ID. The 3-month group was used as the baseline comparison group (indicated by *). Estimate values for older cohorts represent the deviation from the 3-month baseline estimate. Alpha was set at 0.05, significant values are bolded. Graphical representations of data are shown in Supplementary Fig S13

Cortex																					
*Predictors*	**Area**				**Branches**									**Mean branch length (um)**					**Soma area**		
	*Estimates*	*CI*	*p*		*Estimates*			*CI*			*p*			*Estimates*		*CI*		*p*	*Estimates*	*CI*	*p*
3 months*	179.61	143.72–215.50	** < 0.001**		16.57			11.96–21.18			** < 0.001**			25.14		22.37–27.91		** < 0.001**	55.07	47.87–62.27	** < 0.001**
13 months	15.07	−34.08–64.21	0.547		−1.06			−7.37–5.25			0.742			0.83		−2.97–4.62		0.668	16.89	7.03–26.75	**0.001**
15 months	58.77	9.63–107.91	**0.019**		3.69			−2.62–10.00			0.251			−0.43		−4.23–3.36		0.823	14.51	4.65–24.37	**0.004**
18 months	89.62	38.87–140.38	**0.001**		3.26			−3.26–9.78			0.326			−0.09		−4.01–3.83		0.965	39.01	28.83–49.19	** < 0.001**
**Random effects**																					
*σ*^2^	5185.26				86.57									54.26					386.26		
τ_00_	1809.21_ID_				29.76_ID_									8.45_ID_					55.04_ID_		
ICC	0.26				0.26									0.13					0.12		
*N*	30_ID_				30_ID_									30_ID_					30_ID_		
Observations	300				300									300					300		
**Hippocampus**																					
*Predictors*	**Area**				**Branches**								**Mean branch length (um)**						**Soma area**		
	*Estimates*	*CI*		*p*	*Estimates*		*CI*			*p*			*Estimates*			*CI*	*p*		*Estimates*	*CI*	*p*
3 months*	174.81	127.59–222.03		** < 0.001**	14.17		8.99–19.35			** < 0.001**			28.75			23.90–33.59	** < 0.001**		52.86	44.88–60.84	** < 0.001**
13 months	49.93	−12.50–112.36		0.117	5.19		−1.65–12.04			0.137			−2.44			−8.83–3.95	0.453		13.34	2.80–23.88	**0.013**
15 months	52.42	−10.04–114.88		0.100	5.91		−0.94–12.76			0.090			−4.31			−10.71–2.10	0.187		14.14	3.59–24.69	**0.009**
18 months	83.62	19.31–147.92		**0.011**	5.63		−1.42–12.68			0.117			−6.30			−12.88–0.29	0.061		27.09	16.23–37.95	** < 0.001**
**Random effects**																					
*σ*^2^	5226.04				76.81								251.66						269.71		
τ_00_	2920.64_ID_				33.72_ID_								10.75_ID_						71.11_ID_		
ICC	0.36				0.31								0.04						0.21		
*N*	29_ID_				29_ID_								29_ID_						29_ID_		
Observations	288				288								288						288		
**Striatum**																					
*Predictors*	**Area**					**Branches**									**Mean branch length (um)**				**Soma area**		
	*Estimates*	*CI*		*p*		*Estimates*			*CI*			*p*			*Estimates*	*CI*		*p*	*Estimates*	*CI*	*p*
3 months*	162.55	126.50–198.60		** < 0.001**		13.78			10.12–17.45			** < 0.001**			29.40	26.46–32.35		** < 0.001**	49.80	43.74–55.86	** < 0.001**
13 months	27.24	−20.45–74.93		0.262		1.47			−3.38–6.32			0.552			−2.49	−6.39–1.40		0.209	17.22	9.20–25.23	** < 0.001**
15 months	40.23	−7.46–87.92		0.098		3.30			−1.55–8.16			0.181			−6.05	−9.95–−2.15		**0.002**	16.84	8.83–24.85	** < 0.001**
18 months	88.89	39.76–138.02		** < 0.001**		7.25			2.25–12.24			**0.005**			−6.78	−10.80–−2.77		**0.001**	29.89	21.64–38.15	** < 0.001**
**Random effects**																					
*σ*^2^	4006.13					54.54									103.13				257.77		
*τ*_00_	1612.18_ID_					15.37_ID_									3.12_ID_				31.01_ID_		
ICC	0.29					0.22									0.03				0.11		
*N*	29_ID_					29_ID_									29_ID_				29_ID_		
Observations	290					290									290				290

**Table 5:** Linear mixed effects models of Sholl analysis in rat brain tissue. Individual microglia were isolated from each animal (*n* = 10 per region) and images underwent Sholl analysis on ImageJ, as described in Fig. 2, to obtain a metric of branching complexity. Individual cell data were analysed using linear mixed effects modelling within the cortex, hippocampus and striatum. Models contained a fixed effect of age, random intercepts of animal ID and distance from cell body. The 3-month group was used as a baseline comparison group, (indicated by *). Estimate values for older cohorts represent the deviation from the 3-month baseline estimate. Alpha was set at 0.05, significant values are bolded. Graphical representations of data are shown in Supplementary Fig S13

*Predictors*	**Cortex**			**Striatum**			**Hippocampus**		
	*Estimates*	*CI*	*p*	*Estimates*	*CI*	*p*	*Estimates*	*CI*	*p*
3 months*	2.51	1.69–3.33	** < 0.001**	2.11	1.26–2.95	** < 0.001**	2.33	1.37–3.29	** < 0.001**
13 months	−0.19	−0.74–0.36	0.497	0.28	−0.10–0.66	0.151	0.07	−0.35–0.49	0.734
15 months	0.15	−0.40–0.69	0.593	0.26	−0.12–0.64	0.176	0.20	−0.22–0.62	0.355
18 months	0.05	−0.51–0.61	0.864	0.57	0.19–0.96	**0.004**	−0.07	−0.50–0.36	0.756
**Random effects**									
* σ*^2^	2.03			2.10			2.14		
* τ*_00_	0.27_ID_			0.11_ID_			0.14_ID_		
	0.79_Distance_			0.98_Distance_			1.25_Distance_		
ICC	0.34			0.34			0.39		
*N*	6_Distance_			6_Distance_			6_Distance_		
	30_ID_			29_ID_			29_ID_		
Observations	3288			3121			3109		

Compared to baseline (3 months), microglia in the rat cortex had a larger total area at both 15 months (*p* = 0.019) and 18 months (*p* = 0.001). Additionally, at all older timepoints, microglia had increased soma area compared to the 3-month group (13 months (*p* = 0.001); 15 months (*p* = 0.004); 18 months (*p* = < 0.001)). In the hippocampus, microglia demonstrated the same pattern of change as in the cortex, with an increased total area at 18 months (*p* = 0.011), and increased soma area at all older timepoints compared to baseline (13 months (*p* = 0.013); 15 months (*p* = 0.009); 18 months (*p* = < 0.001)). No changes with age in the number of branches or branch length were detected in either the cortex or hippocampus (Table 4). Sholl analysis also demonstrated no change in branching complexity in these regions with age (Table 5).

In the striatum, microglia in rats again showed the same pattern of change in total area and soma area. Microglia within the striatum had increased total area at 18 months compared to 3 months (*p* = < 0.001) and increased soma area at all older timepoints (13 months (*p* = < 0.001); 15 months (*p* = < 0.001); 18 months (*p* = < 0.001)). Additionally, microglia in the striatum had an increased number of branches at 18 months (*p* = 0.005) but showed a progressive decrease in mean branch length with age, which was significantly reduced compared to 3 months at both 15 months (*p* = 0.002) and 18 months (*p* = 0.001). Furthermore, at 18 months, microglia in the striatum showed an increase in branching complexity compared to 3 months (*p* = 0.004). Graphical representations of all morphological data from rat analyses are presented in Supplementary Figure S13.

### Microglial morphology in the sheep brain is only altered with age in the striatum, with distinct patterns seen in the caudate versus the putamen

Linear mixed effects models were employed to investigate the effect of age on microglial morphology in sheep tissue, with results from models presented in Table 6 for area and skeletal analyses and Table 7 for Sholl analysis. These models used the 1–1.5-year group as a baseline category and included a fixed effect of age and a random intercept for animal ID. In Sholl analysis, distance, operationalised as increments of 10 µm, was included as an additional random intercept. For ease of reporting, estimates and confidence intervals are presented only in the relevant tables.

**Table 6:** Linear mixed effects modelling of microglial morphological analyses in sheep brain tissue. Individual microglia were isolated from each animal (*n* = 10 per region) and analysed on ImageJ, as described in Fig. 2, to obtain cell area, number of branches, mean branch length per cell and soma area. Individual cell data were analysed using linear mixed effects modelling within the cortex, DG, HP, CN and PUT. Models contained a fixed effect of age, and a random intercept of animal ID. The 1–1.5-year group was used as a baseline comparison group, (indicated by *). Estimate values for older cohorts represent the deviation from the 1–1.5-year baseline estimate. Alpha was set at 0.05, significant values are bolded. Graphical representations of data are shown in Supplementary Fig S14

Cortex																									
*Predictors*	**Area**			**Number of branches**						**Mean branch length (µm)**								**Soma area**							
	*Estimates*	*CI*	*p*	*Estimates*		*CI*	*p*			*Estimates*		*CI*			*p*			*Estimates*			*CI*			*p*	
1–1.5 years*	144.12	131.12–156.93	** < 0.001**	9.57		8.24–10.90	** < 0.001**			8.45		7.55–9.35			** < 0.001**			70.77			60.28–81.27			** < 0.001**	
3–3.5 years	1.18	−17.09–19.46	0.899	0.12		−1.79–2.02	0.902			0.70		−0.59–1.99			0.285			7.08			−7.46–21.62			0.339	
5–6 years	17.55	−5.68–40.79	0.138	−0.07		−2.49–2.35	0.954			0.83		−0.80–2.46			0.315			8.83			−10.55–28.21			0.371	
**Random effects**																									
σ^2^	2745.95			33.65						26.28								381.72							
τ_00_	405.72_ID_			4.00_ID_						0.71_ID_								442.42_ID_							
ICC	0.13			0.11						0.03								0.54							
N	38_ID_			38_ID_						38_ID_								38_ID_							
Observations	380			380						380								380							
**DG**																									
*Predictors*	**Area**			**Number of branches**							**Mean branch length (µm)**								**Soma area**						
	*Estimates*	*CI*	*p*	*Estimates*	*CI*			*p*			*Estimates*		*CI*			*p*			*Estimates*			*CI*		*p*	
1–1.5 years*	164.87	149.49–180.25	** < 0.001**	15.14	13.23–17.04			** < 0.001**			7.63		6.77–8.48			** < 0.001**			64.66			59.46–69.86		** < 0.001**	
3–3.5 years	4.03	−17.33–25.39	0.711	1.51	−1.14–4.16			0.262			0.01		−1.17–1.20			0.983			−1.09			−8.31–6.13		0.766	
5–6 years	17.09	−7.78–41.96	0.177	1.32	−1.76–4.40			0.401			−0.29		−1.68–1.09			0.676			2.06			−6.34–10.47		0.629	
**Random effects**																									
* σ*^2^	4378.09			84.29							19.58								325.09						
* τ*_00_	357.35_ID_			3.78_ID_							0.50_ID_								58.25_ID_						
ICC	0.08			0.04							0.02								0.15						
*N*	35_ID_			35_ID_							35_ID_								35_ID_						
Observations	351			351							351								351						
**HP**																									
*Predictors*	**Area**			**Number of branches**							**Mean branch length (µm)**								**Soma area**						
	*Estimates*	*CI*	*p*	*Estimates*	*CI*			*p*			*Estimates*		*CI*			*p*			*Estimates*			*CI*		*p*	
1–1.5 years*	177.48	159.47–195.49	** < 0.001**	16.32	13.74–18.90			** < 0.001**			7.48		6.88–8.08			** < 0.001**			67.75			63.30–72.20		** < 0.001**	
3–3.5 years	1.21	−22.29–24.70	0.919	0.77	−2.55–4.09			0.647			−0.01		−0.81–0.78			0.975			1.12			−4.77–7.00		0.710	
5–6 years	9.14	−19.12–37.40	0.525	0.44	−3.59–4.46			0.831			0.01		−0.94–0.96			0.989			3.11			−3.91–10.13		0.384	
**Random effects**																									
*σ*^2^	4476.58			80.25							5.98								314.18						
*τ*_00_	767.36_ID_			17.66_ID_							0.71_ID_								40.83_ID_						
ICC	0.15			0.18							0.11								0.12						
*N*	35_ID_			35_ID_							35_ID_								35_ID_						
Observations	350			350							350								350						
**CN**																									
*Predictors*	**Area**			**Number of branches**							**Mean branch length (µm)**									**Soma area**					
	*Estimates*	*CI*	*p*	*Estimates*		*CI*			*p*		*Estimates*			*CI*			*p*			*Estimates*			*CI*		*p*
1–1.5 years*	178.99	122.01–235.98	** < 0.001**	17.73		15.01–20.46			** < 0.001**		6.63			6.02–7.24			** < 0.001**			68.39			0.55–136.22		**0.048**
3–3.5 years	−9.22	−91.14–72.70	0.825	0.57		−3.35–4.49			0.775		−0.62			−1.49–0.26			0.166			−1.62			−99.14–95.90		0.974
5–6 years	120.24	16.94–223.53	**0.023**	−0.16		−5.10–4.78			0.949		−0.01			−1.12–1.09			0.981			127.92			4.95–250.88		**0.042**
**Random effects**																									
*σ*^2^	134,381.41			77.22							6.22									190,432.37					
*τ*_00_	0.00_ID_			23.01_ID_							0.91_ID_									0.00_ID_					
ICC				0.23							0.13														
*N*	38_ID_			38_ID_							38_ID_									38_ID_					
Observations	380			380							380									380					
**PUT**																									
*Predictors*	**Area**			**Number of branches**							**Mean branch length (µm)**								**Soma area**						
	*Estimates*	*CI*	*p*	*Estimates*	*CI*			*p*			*Estimates*		*CI*			*p*			*Estimates*			*CI*		*p*	
1–1.5 years*	163.29	150.51–176.07	** < 0.001**	16.39	14.13–18.64			** < 0.001**			6.54		5.94–7.13			** < 0.001**			64.10			59.55–68.65		** < 0.001**	
3–3.5 years	−18.61	−36.98–−0.24	**0.047**	−3.35	−6.59–−0.11			**0.043**			0.50		−0.35–1.35			0.247			2.84			−3.71–9.38		0.394	
5–6 years	18.32	−4.85–41.48	0.121	−1.96	−6.05–2.13			0.347			1.24		0.17–2.31			**0.023**			8.72			0.47–16.97		**0.038**	
**Random effects**																									
*σ*^2^	3020.23			61.60							8.62								236.68						
*τ*_00_	373.77_ID_			14.89_ID_							0.58_ID_								62.03_ID_						
ICC	0.11			0.19							0.06								0.21						
*N*	38_ID_			38_ID_							38_ID_								38_ID_						
Observations	380			380							380								380

**Table 7:** Linear mixed effects models of Sholl analysis in sheep brain tissue. Individual microglia were isolated from each animal (*n* = 10 per region) and images underwent Sholl analysis on ImageJ, as described in Fig. 2, to obtain a metric of branching complexity. Individual cell data were analysed using linear mixed effects modelling within the cortex, DG, HP, CN and PUT. Models contained a fixed effect of age, random intercepts of animal ID and distance from cell body. The 1–1.5-year group was used as a baseline comparison group, (indicated by *). Estimate values for older cohorts represent the deviation from the 1–1.5-year baseline estimate. Alpha was set at 0.05, significant values are bolded. Graphical representations of data are shown in Supplementary Fig S14

*Predictors*	**Cortex**			**DG**			**HP**			**CN**			**PUT**		
	*Estimates*	*CI*	*p*	*Estimates*	*CI*	*p*	*Estimates*	*CI*	*p*	*Estimates*	*CI*	*p*	*Estimates*	*CI*	*p*
1–1.5 years*	1.05	0.01–2.08	**0.047**	0.98	0.09–1.88	**0.032**	1.24	0.25–2.23	**0.014**	1.06	−0.19–2.30	0.096	1.04	−0.16–2.25	0.090
3–3.5 years	0.01	−0.25–0.27	0.940	0.06	−0.18–0.30	0.636	−0.06	−0.40–0.28	0.736	0.01	−0.35–0.37	0.954	0.27	−0.06–0.60	0.112
5–6 years	−0.06	−0.26–0.15	0.579	−0.20	−0.41–0.01	0.057	−0.23	−0.52–0.07	0.135	0.09	−0.20–0.37	0.552	0.25	−0.01–0.51	0.060
Random effects															
*σ*^2^	1.28			1.53			1.69			1.72			1.59		
*τ*_00_	0.08_ID_			0.07_ID_			0.14_ID_			0.15_ID_			0.12_ID_		
	1.36_Radius_			1.62_Radius_			1.70_Radius_			2.35_Radius_			1.85_Radius_		
ICC	0.53			0.52			0.52			0.59			0.55		
*N*	5_Radius_			8_Radius_			7_Radius_			6_Radius_			5_Radius_		
	38_ID_			35_ID_			35_ID_			38_ID_			38_ID_		
Observations	5172			6371			6474			6030			5538		

No significant changes in total microglial area, soma area, number of branches or branch length were observed in the cortex, DG or HP (Table 6). Similarly, there were no differences in branching complexity in any of these regions (Table 7). There were, however, changes in microglial morphology within the striatum. In the CN, microglia had a larger cell area and soma area in the 5–6-year group compared to the 1–1.5-year group (cell area: (*p* = 0.023) soma area: (*p* = 0.042)), but no significant differences were seen between groups for either number of branches or branch length. Within the PUT, microglia at 3–3.5 years were smaller (*p* = 0.047) and had fewer branches (*p* = 0.043) than microglia at 1–1.5 years. However, at 5–6 years, both microglial size (*p* = 0.121) and number of branches (*p* = 0.347) were unchanged compared to 1–1.5-year animals. Microglia in this group did, however, have an increased branch length (*p* = 0.023) and soma area (*p* = 0.038) compared to 1–1.5-year animals. Graphical representations of sheep morphological data are shown in Supplementary figure S14. Table 8 summarises the observed changes from baseline (youngest age group) for each species at each age.

**Table 8.**
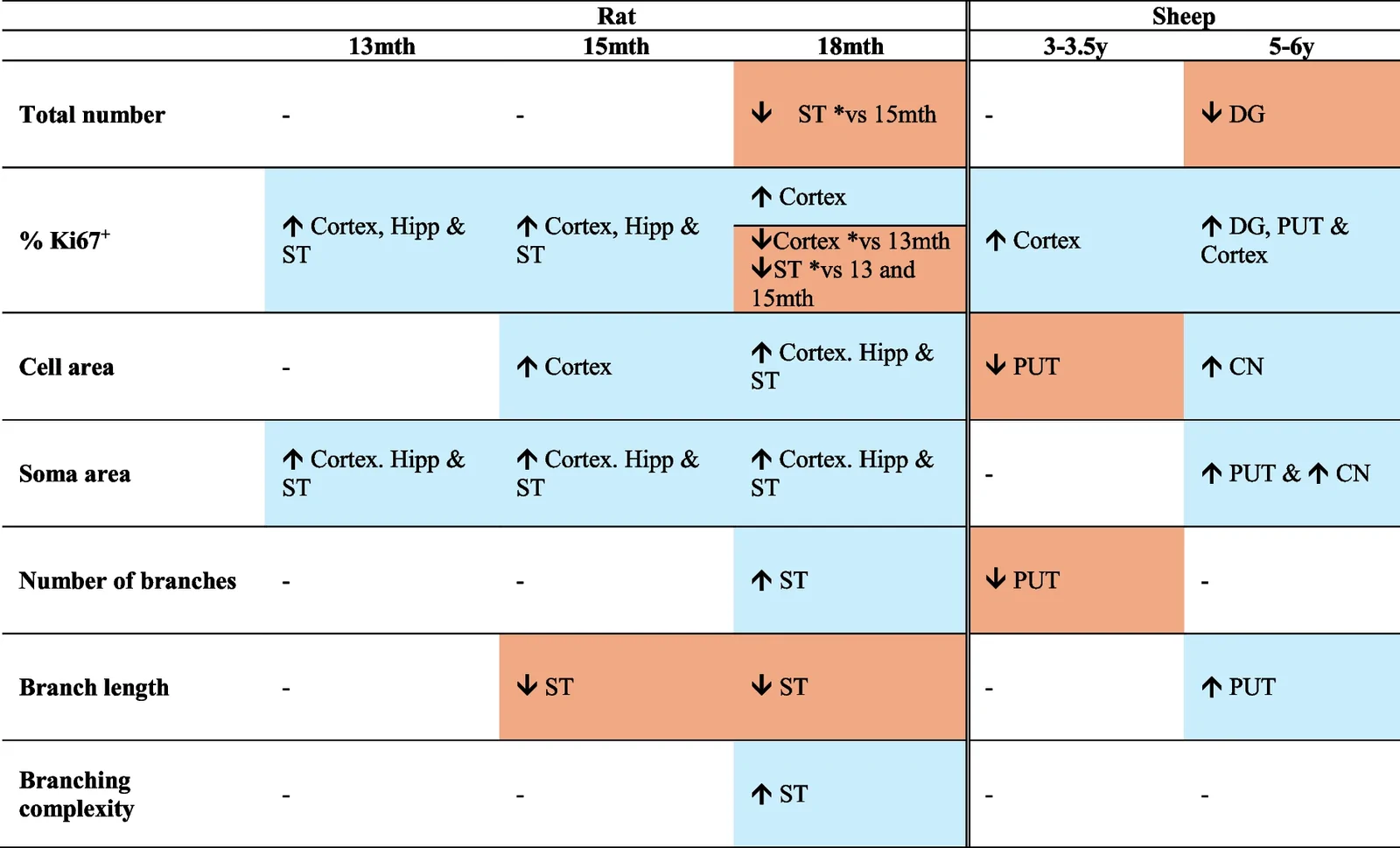
Changes observed in microglia in the current study. Significant changes between age groups for each marker assessed are summarised below. Changes listed are in reference to the youngest age group for that species (3 months in rats, 1–1.5 years in sheep) except for where specified with *. *Hipp*, hippocampus, *ST*, striatum, blue shading with a **á** indicates an increase, orange shading with a **â** indicates a decrease, and no shading with a dash (-) indicates no change from the youngest age group

## Discussion

The current study represents, to the best of our knowledge, the first cross-species comparison of changes in microglial population and morphology with age across multiple brain regions in a naïve lissencephalic and a large gyrencephalic species. Manual quantification of microglia revealed a similar regional pattern of results in rats and sheep, with a marked increase in proliferation across the lifespan in the cortex and in regions of the hippocampus (DG) and striatum (PUT) in both species. Interestingly, this proliferation began to decline at the oldest timepoint assessed (i.e. 18 months old) in rats, while there was instead an increase in proliferation in sheep. Total microglial number was largely unchanged with age in the rat brain, except for a slight decrease at the oldest timepoint assessed. Similarly, in sheep, there was a significant decrease in the number of microglia in the DG in the oldest sheep, with a non-statistically significant decrease also seen at this timepoint in the striatum and cortex. Morphometric analysis showed increased size at the oldest time points in both species. However, while these changes in cell and soma size were widespread in the rat brain, in sheep, they were localised only to the striatum. Furthermore, changes in microglial branching occurred exclusively in the striatum in both species. Taken together, these results implicate the striatum as a potential early site of age-related microglial change, with the numbers of microglia decreasing with age in the late middle adulthood period, but the remaining cells becoming larger and, at least in rats, more ramified.

### Consideration of the age of animals used in the current study

**Fig. 5:**
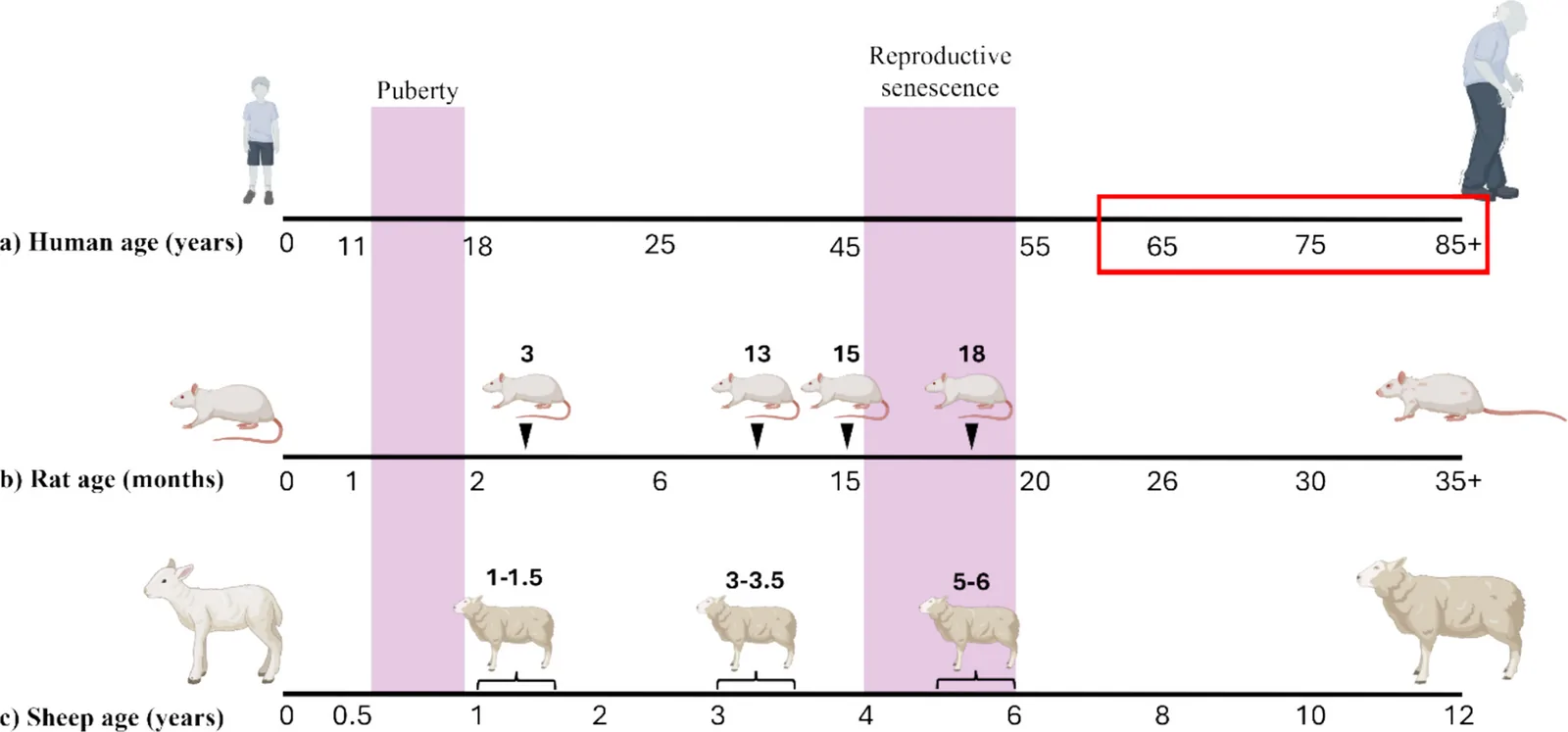
Visual representation of **a** human ageing compared to the pre-clinical animal models used in the current study; the red box indicates the age at which neurodegenerative diseases most often emerge in humans. Comparative ages are estimates based on female reproductive milestones in each species. Ages utilised in the current cohort are indicated for **b** rats and **c** sheep

Prior to considering our results in detail, it is important to understand how the ages of animals assessed in the current study compare to humans, as this has significant implications for translational relevance. While human age is not linear with rat or sheep age, due to the different rates of development between species [63], approximate equivalent ages can be discerned based on female reproductive milestones. Figure 5 displays an estimation of comparable ages across species based on these milestones in humans [64], rats [63] and sheep [65, 66]. Based on these estimates, the 18-month-old rats and 5–6-year-old sheep used in the current study were roughly equivalent to 55-year-old humans. Conversely, the 13-month rats and 3–3.5-year-old sheep were roughly equivalent to a human in their late 20 s or early 30 s.

Thus, the latest timepoints assessed in the current study correspond to late middle age in humans, rather than a true aged cohort. Therefore, findings in the present study likely reflect an age at which ageing processes are beginning to gradually occur, prior to the time at which age-related neurological diseases would be expected to present (i.e. 55 + years of age). This is significant, as such diseases are known to be associated with brain changes for years, or even decades, prior to symptom onset. For example, in Parkinson’s disease, which involves significant alterations in nigrostriatal circuitry, the prodromal phase can last up to 20 years, with evidence that approximately 11% of the late middle-aged population presents with two or more known risk factors for Parkinson’s disease [67]. Thus, studies that assess species-specific differences across adulthood, such as this one, have translational relevance for understanding how changes in specific neural cell populations evolve over time. Such foundational knowledge may allow for early identification of biomarkers of risk of development of age-related neurological disease.

### Elevated proliferation rate largely maintains homeostasis of total microglial population into middle adulthood

An increase in expression of markers of microglial proliferation was observed in all regions of the rat brain at the middle timepoints, as well as within the cortex, DG and PUT in sheep. These findings are consistent with prior reports in the literature that microglia in the adult rodent and human brain display a high proliferation rate, a process temporally and spatially coupled to intrinsic apoptosis, which allows for dynamic and highly regulated control of a relatively stable number of total microglial throughout the lifespan [37]. In line with this, in previous reports using microglia isolated from the cortex of F344 rats, a steady increase in microglial proliferation in culture, as measured by [3H]-Thymidine labelling, was seen across adulthood [68]. Interestingly, in the current study, at 18 months, this microglial proliferation appeared to slightly decrease or stabilise to levels observed at the middle timepoints in our rat cohort. This suggests that microglial proliferation rate may begin to taper off with increased age, at least within some regions, making it potentially more difficult to maintain homeostasis of the total microglial population.

In contrast, however, an earlier study investigating microglia isolated from the cortex of F344 rats found no decreases in proliferation at older time periods, with an up to 400% increase in proliferation in microglia isolated from 24-month-old rats compared to those from 3-month-old rats [68]. Similarly, increases in proliferation were seen across multiple regions at the oldest timepoint assessed in sheep in the current study (although further increases were not seen in 5–6-year-old sheep relative to 3–3.5-year-old sheep). It is also important to note that both our results and those of the previous study in F344 rat microglia contrast with the pattern seen in a human post-mortem study, where no changes in Ki67-positive microglia across adulthood were observed (58–79 years old compared to 20–35 years old) [37]. This may suggest potential species- or strain-specific disparity in microglial proliferation rates, but this needs to be further probed using more sensitive measures of cell turnover and with a focus on the implications of changes in proliferation for microglial function. For example, recent work using RNA-Sequencing has demonstrated that proliferating microglia have unique transcriptomic signatures that change both with age and in a neurodegenerative environment [69].

Interestingly, in the current study, while markers of proliferation showed significant increases throughout adulthood in both species, the total number of microglia was relatively stable across time, with a few exceptions. Specifically, there was a decrease in the total number of cells within the striatum of 18-month rats compared to 15-month rats and a significant decrease with age in the DG of 5–6-year sheep. This is largely consistent with previous reports of consistent microglial turnover (approximately 28% per year in humans) [70], but with an overall stable population across the lifespan [37, 38]. This homeostatic balance is achieved through rapid and continuous turnover of microglia, with a balanced coupling of proliferation and intrinsic apoptosis [37]. In fact, studies in both the human and rodent brain have demonstrated that the microglial population turns over several times during a lifetime [37], which contrasts with earlier theories that the adult microglial adult population is long-lived, with maintenance through self-renewal [71].

Nevertheless, not all literature is consistent, with a few prior studies reporting decreases in microglial number with age [72, 73]- similar to the relatively minor reductions observed here- and even more reporting increases. For example, increases in microglial number with age have been reported in the auditory cortex of Sprague–Dawley rats [74], the amygdala of F344 rats [51], subcortical [73] and cortical regions of C57BL6 mice [72], and in the cortex in post-mortem human tissue [75]. Given these discrepancies, further investigation of microglial alterations across the lifespan, particularly at older timepoints, as well as across different species, is needed. It is also important to investigate what may drive such alterations in microglial number. For example, although significant decreases in the DG and non-statistically significant decreases in the cortex and stratum were observed in the total number of microglia for the oldest age group of sheep in the current work, this was not associated with a concomitant reduction in proliferation rate within these regions. Future studies should therefore investigate the biological mechanisms that contribute to alterations in homeostatic balance in microglial number with age, beyond proliferation rate alone.

### Morphometric changes in microglia differ across species, but are present mainly in the striatum

While microglia were once thought to be generally similar throughout the CNS, more recent reports have challenged this understanding. Specifically, microglia in young adult mice have been shown to display regionally distinct phenotypes [76–78], even under homeostatic conditions [79]. Importantly, microglia have recently been demonstrated to show regional-specific responses to ageing, even as early as middle age [80]. To probe this further, we investigated morphological changes in microglia across multiple brain regions up to late middle adulthood.

In rats, multiple brain regions demonstrated increases in cell and soma area at the oldest timepoint; however, in sheep, this was localised to the striatum. This is consistent with a previous report of increased microglial soma area in multiple brain regions in 18-month-old male and female F344 rats, including the bed nucleus of stria terminalis, medial amygdala and CA3 region [51]. This microglial hypertrophy largely aligns with the traditional concept of the activated microglial phenotype and such a morphological shift has been widely reported to occur with age (for review, see [81, 82]). It is important to note, however, that the idea of a single activated microglial phenotype is no longer widely accepted in the literature [82].

In addition to these size changes, an increase in both branch number and branching complexity, but a decrease in branch length, was observed exclusively in the striatum in 18-month-old rats. This is in line with the results of a previous study, which demonstrated that, at 27 months old, microglia in the striatum of C57BL/6 J mice show pronounced hypertrophy and shorter and thicker processes [83]. Prior postmortem studies in the human brain have also demonstrated that microglial cell processes become shorter, with less branching and reduced arborisation, with normal physiological ageing [84]. Therefore, the increase in cell and soma size and decrease in branch length seen here in older animals was not surprising; however, the increase in branch number and complexity was less expected. Additionally, we observed an increase in branch length in the putamen of 5–6-year-old sheep relative to 1–1.5-year-old sheep which contrasts with the typical picture of aged microglia as amoeboid cells [82]. As discussed above, however, the oldest animals assessed here do not represent a true aged population, but rather late middle adulthood, and therefore these changes may instead illustrate the dynamic nature of microglia throughout the lifespan, as they participate in multiple functions ranging from immune surveillance to regulation of synapses and neuronal activity [85]. Thus, future work should address how alterations in microglial morphology continue to evolve over time in older adult animals.

Interestingly, in our study, changes in microglial branching in rats were only present in the striatum, and were not observed in other regions, even within the hippocampus, where changes have been reported in earlier rodent work [50]. While this may be due to strain differences (i.e. Wistar versus Sprague–Dawley rats), it is also possible that the hippocampus may be a site of later morphological change, as compared to the striatum, a region not often assessed in ageing studies [50]. Our results in sheep further corroborate the apparent vulnerability of the striatum to the effects of age, as this region was the only area where morphometric changes were observed. This suggests that the striatum is a region of particular importance when it comes to microglial ageing across multiple species. Indeed, a variety of studies have supported that the striatum undergoes significant age-related change, even in healthy adults [86, 87]. When using MRI to measure the volume of structures of the striatum in 53 healthy adults with a baseline age of 20 to 77 years old, Raz et al. (2003) found that, over 5 years, regions of the striatum showed progressive atrophy, with an annual decline in in volume of 0.5–0.8% depending on the region [86]. Furthermore, recent work using single-nucleus RNA sequencing has demonstrated that striatal microglia show a distinct molecular signature of stress, with greater estimated microglial maturity apparent even at 42 days following a stressor exposure [88].

This unique early vulnerability of striatal microglia to the effects of ageing may have implications for neurodegenerative conditions that preferentially impact the striatum, such as Huntington’s disease [89] or Parkinson’s disease [90]. Age-related microglial alterations in the striatum may prime the region for dysfunction and potentially exacerbate neuronal vulnerability, ultimately contributing to the emergence of disease pathology. Further research should investigate how these underlying morphological changes correlate with microglial function and how they impact regional vulnerability of the striatum. This is particularly important given the timing of the changes observed here, as the middle-aged period represents a time when disease pathology begins to emerge in the decades prior to diagnosis, representing a critical period for early risk identification [91].

## Limitations and future considerations

Given that many age-related disorders do not manifest clinically until 55 + years of age, or even later [92], the inability to probe age-related changes in microglia in older adulthood is a limitation of the current work. While this is mitigated somewhat by the fact that brain changes in age-related neurological disease are known to occur for decades prior to symptom emergence [93, 94], future studies should nevertheless seek to include older animals, in order to more fully investigate the time course of microglial change across the lifespan.

It must be acknowledged, however, that conducting studies in older animals is not without significant challenges. The difficulties in sourcing, housing and caring for aged animals, particularly in facilities without ready access to colonies of older animals or without specialised experience in maintaining aged colonies, are one of the biggest barriers to including age in current small animal studies [8, 95]. This becomes even more challenging in large animals due to animal size, longer lifespans and increased costs [18]. The Aging Rodent Colonies project at the National Institute on Aging [95] has somewhat aided in this effort by supplying aged rodents for National Institute of Health (NIH) funded aging research [95]. However, for those located outside the United States or funded by non-NIH sources, funding for and access to aged animals is still a major concern. Additionally, healthcare challenges seen in aged animals, large or small, differ greatly from those of young animals [96–101]. Researchers planning ageing studies therefore need to be adequately prepared to cater for these unique challenges.

Samples from previous ageing studies are therefore highly valuable and allow for additional analyses beyond the original research question(s) to be investigated using archival tissue, such as was done in the current study. This helps to reduce the need to generate older animal cohorts, also helping to reduce the excessive use of animals in research. However, use of archival tissue is not without limitations. In the case of the current study, the age of the animals limited the ability to draw conclusions beyond the late middle age period. Furthermore, within the rat cohort, we were limited to archival tissue from male animals and thus could not assess any sex-specific differences. This is a potentially significant gap, given that investigations in human post-mortem tissue [75] and in rats [51] have demonstrated sex differences in microglia. While the present study did have tissue from both males and females available for the sheep cohort, numbers were imbalanced between age groups. Most notably, the 5–6-year group was exclusively female (0 M;8F), which may have influenced our results. Analysis and graphical representation of the sheep data stratified into males and females is presented in Supplementary Table S1 and Supplementary Fig S1; however, due to the aforementioned imbalance of sex in the oldest age group, it was not possible to fully assess the impact of sex on age-related changes in microglia.

## Conclusions

The current study contributes to the growing body of literature on the shift that microglia experience with age (for a recent review on this topic by our group, see [82]) by presenting a cross-species comparison of microglial alterations across the lifespan in diverse regions of the rat and sheep brain. Importantly, we demonstrated similar regional patterns of change across species in terms of both when and where microglia begin to display distinct morphological changes consistent with ageing. We also highlight the striatum as a region in which microglia are particularly vulnerable to such changes. Understanding how microglial phenotype evolves across adulthood provides foundational knowledge that can inform the timing of targeting aberrant neuroinflammation for the treatment of age-related neurological disease. Tracking such changes in middle adulthood may also have utility for identifying risk of later age-related neurological disease.

Furthermore, data from the current study emphasise the critical importance of incorporating age into preclinical modelling, despite the financial and practical difficulties involved. Future work should seek to characterise microglial changes seen in animals used for preclinical modelling of neurological disease over a longer period of time and, importantly, assess how this compares to the changes seen in both healthy ageing and neurological disease in human, as well as whether this is influenced by biological sex. As microglia do not function in isolation, age associated changes in other glial cells, including astrocytes [102] and oligodendrocytes [103], as well as in neurons themselves [104], should also be investigated, in order to give a more complete picture of the cellular-level changes that occur across the lifespan. These comparisons will further elucidate the role of ageing in the brain’s susceptibility to neurological disease and enhance our understanding of how such changes impact treatment outcomes.

## Supplementary Information

Below is the link to the electronic supplementary material.

ESM1 (DOCX 21.1 MB)

## Data Availability

Data supporting the findings of this study are available from the corresponding author upon reasonable request.
