# Supplementary material for: Cross-species characterisation of microglial morphology and proliferation during ageing
Source: GeroScience. 2026 Apr 24;48(4):5109–33. doi: 10.1007/s11357-026-02254-3 (PMC13575081; doi:10.1007/s11357-026-02254-3)
Supplement: Supplementary file 1 [file 11357_2026_2254_MOESM1_ESM.docx]

**Supporting Information for**

Cross-Species Characterisation of Microglial Morphology and Proliferation During Ageing

Laura Carr^1^, Angus McNamara^1^, Shannon M Stuckey^1^, Isabella M Bilecki^1^, Sanam Mustafa^1,2,3^, Renée J Turner^1^, Lyndsey E Collins-Praino^1*^

^1^School of Biomedicine, University of Adelaide, Adelaide, Australia

^2^Davies Livestock Research Centre, University of Adelaide, Roseworthy, SA, Australia

^3^Australian Research Council Centre of Excellence for Nanoscale Biophotonics, The University of Adelaide, Adelaide, SA, Australia

A/Professor Lyndsey Collins-Praino

Email: [Lyndsey.collins-praino@adelaide.edu.au](mailto:Lyndsey.collins-praino@adelaide.edu.au)

**This document includes**

Supplementary Figures S1-S13

Supplementary Table S1

Supplementary Figure S1: Microglia in the sheep brain, grouped by age and sex. a) total number of microglia detected by manual counting. n = 1-1.5y (8M:8F), 3-3.5y (8M:8F), 5-6y (0M:8F) b) number of Ki67+ microglia detected by manual counting. n = 1-1.5y (3M:5F), 3-3.5y (5M:3F), 5-6y (0M:8F). Data are graphed as mean±SEM. Significant differences were determined by two-way ANOVA (results presented in table S1). * = p<0.05, ** = p<0.01

**a.**

**b.**

**c.**

**d.**

**a.**

Supplementary Figure S2: Representative levels and images of area analysed for rat and sheep tissue. Levels for analysis were obtained for rats using the rat brain atlas [47] and sheep using the sheep brain atlas, available from Michigan State University website [48]. In rat tissue, the whole annotated area in the striatum (a.) and hippocampus (b.) were counted. The hippocampus in both hemispheres was included across two serial sections of tissue. For the cortex, boxes were drawn at both levels for analysis. In sheep, boxes were drawn to denote analysis regions for the cortex (c. cyan outline), caudate (c. purple outline), putamen (c. green outline) and hippocampus proper (d. purple outline). The entire area of the dentate gyrus (d. red outline) was included for analysis.

Supplementary Figure S3: Representative images of immunofluorescent staining in aged rat (18-mo) and sheep (1-1.5 year) brains in hippocampal and striatal regions. Microglia were counted by identifying colocalised DAPI and IBA1. Microglia were classified as Ki67^+^ when both DAPI, IBA1 and Ki67 signal was present. The number of Ki67^+^ microglia were counted manually and analysed as a percentage of the total microglial population. Scale bar = 100µm

Supplementary Figure S4: Percentage of cell positive for Ki67 in the rat striatum before outlier removal using ROUT (Q=1%). One outlier was removed from the 18mth group (value =7.5%). The outcome of the ANOVA was not altered *(F(3,25)=11.30, p=<0.0001)* however the strength of the post-hoc test was changed for the 13mth vs 18mth comparison *(7.944±1.48 vs 1.997±1.01, p= 0.007*

Supplementary Figure S5: Individual channels for representative images in Fig 3a. Microglia were stained for IBA1 (red) in the rat cortex at each age. IBA1^+^ cells with a strong Ki67 signal (green) were classified as Ki67^+^ microglia. Nuclei were visualized using DAPI (blue). Scale bar = 100µm

Supplementary Figure S6: Individual channels for representative images in Fig 3a. Microglia were stained for IBA1 (red) in the rat Hippocampus at each age. IBA1^+^ cells with a strong Ki67 signal (green) were classified as Ki67^+^ microglia. Nuclei were visualized using DAPI (blue). Scale bar = 100µm

Supplementary Figure S7: Individual channels for representative images in Fig 3a. Microglia were stained for IBA1 (red) in the rat Striatum at each age. IBA1^+^ cells with a strong Ki67 signal (green) were classified as Ki67^+^ microglia. Nuclei were visualized using DAPI (blue). Scale bar = 100µm

Supplementary Figure S8: Individual channels for representative images in Fig 4a. Microglia were stained for IBA1 (red) in the sheep cortex at each age. IBA1^+^ cells with a strong Ki67 signal (green) were classified as Ki67^+^ microglia. Nuclei were visualized using DAPI (blue). Scale bar = 100µm

Supplementary Figure S9: Individual channels for representative images in Fig 4a. Microglia were stained for IBA1 (red) in the sheep Dentate Gyrus at each age. IBA1^+^ cells with a strong Ki67 signal (green) were classified as Ki67^+^ microglia. Nuclei were visualized using DAPI (blue). Scale bar = 100µm

Supplementary Figure S10: Individual channels for representative images in Fig 4a. Microglia were stained for IBA1 (red) in the sheep hippocampus proper at each age. IBA1^+^ cells with a strong Ki67 signal (green) were classified as Ki67^+^ microglia. Nuclei were visualized using DAPI (blue). Scale bar = 100µm

Supplementary Figure S11: Individual channels for representative images in Fig 4a. Microglia were stained for IBA1 (red) in the sheep caudate nucleus at each age. IBA1^+^ cells with a strong Ki67 signal (green) were classified as Ki67^+^ microglia. Nuclei were visualized using DAPI (blue). Scale bar = 100µm

Supplementary Figure S12: Individual channels for representative images in Fig 4a. Microglia were stained for IBA1 (red) in the sheep putamen at each age. IBA1^+^ cells with a strong Ki67 signal (green) were classified as Ki67^+^ microglia. Nuclei were visualized using DAPI (blue). Scale bar = 100µm

Supplementary Figure S13: Graphical representation of rat morphological analysis. Individual microglia were isolated from each animal (n=10 per region) and images underwent analysis on ImageJ to obtain a metric of branching complexity. Individual cell data were analysed using linear mixed effects modelling within ROIs and results are presented in the main text. Models contained a fixed effect of age and a random intercept of animal ID. For Sholl analysis, models also contained a random intercept of distance from cell body. The 3-month group was used as a baseline comparison group; alpha was set at 0.05. * = p<0.05, ** = p<0.01, *** = p<0.001

Supplementary Figure S14: Graphical representation of sheep morphological analysis. Individual microglia were isolated from each animal (n=10 per region) and images underwent analysis on ImageJ to obtain a metric of branching complexity. Individual cell data were analysed using linear mixed effects modelling within ROIs and results are presented in the main text. Models contained a fixed effect of age and a random intercept of animal ID. For Sholl analysis, models also contained a random intercept of distance from cell body. The 1-1.5 year group was used as a baseline comparison group, alpha was set at 0.05. * = p<0.05.

Supplementary Table S1: Results from two-way ANOVA conducted on sheep microglia data stratified into male and female groups. For total counts significant main effects of sex were seen in the DG and HP. For Ki67 counts an effect of sex was seen in the PUT. Due to the low absence of males in the aged cohort interaction effects could not be assessed.

|  |  | DG | HP | CN | PUT | Cortex |
| --- | --- | --- | --- | --- | --- | --- |
| Total | Age | **F (2, 29) = 17.39,**  **P=<0.0001** | **F (2, 30) = 5.556,**  **P=0.008** | **F (2, 33) = 5.599,**  **P=0.008** | **F (2, 34) = 4.248,**  **P=0.022** | **F (2, 34) = 5.726,**  **P=0.007** |
|  | Sex | **F (1, 29) = 31.07,**  **P=<0.0001** | **F (1, 30) = 10.73,**  **P=0.002** | F (1, 33) = 2.604,  P=0.116 | F (1, 34) = 2.071,  P=0.159 | F (1, 34) = 3.123,  P=0.086 |
| Ki67+ | Age | **F (2, 20) = 4.453,**  **P=0.0251** | F (2, 20) = 1.572,  P=0.2321 | F (2, 17) = 3.244,  P=0.0640 | **F (2, 19) = 4.896,**  **P=0.0193** | **F (2, 20) = 5.688,**  **P=0.0111** |
|  | Sex | F (1, 20) = 0.3375,  P=0.5678 | F (1, 20) = 0.5651,  P=0.4610 | F (1, 17) = 0.6135,  P=0.4443 | **F (1, 19) = 5.063,**  **P=0.0365** | F (1, 20) = 0.5948,  P=0.4496 |
